# Impact of sex-based and sexual orientation-based victimization on training discontinuity among swiss apprentices: a longitudinal study

**DOI:** 10.1186/s40461-026-00211-0

**Published:** 2026-06-20

**Authors:** Lavinia Gianettoni, Jérôme Blondé, Edith Guilley, Matteo Lacalamita

**Affiliations:** 1 University of Lausanne, Lausanne, Switzerland; 2 University of Geneva, Geneva, Switzerland; 3 Service de la recherche en éducation, Geneva, Switzerland

**Keywords:** Training discontinuity, Sex-based victimization, Sexual orientation-based victimization, Vocational education and training, Longitudinal research, Concealment of sexual orientation

## Abstract

This longitudinal study examines the impact of sex-based and sexual orientation-based victimization on training discontinuity among students in the Swiss Vocational Education and Training (VET) system. Drawing on a representative cohort of 1370 first-year apprentices enrolled in six vocational training centers in the canton of Geneva, data were collected across three waves (2020–2023). Experienced violence was measured at three time points, and training discontinuity was defined as absence from class registers in the subsequent academic year (T + 1). Logistic regression models were estimated on 15 multiply imputed datasets to address missing data. Results indicate that experiencing sexual orientation-based violence at the beginning of the apprenticeship (T1) significantly increases the likelihood of training discontinuity at T2, even after controlling for assigned sex, the sex-typing of training fields, and concealment of sexual orientation. In contrast, sex-based violence was not significantly associated with training discontinuity in the following year, and sexual orientation-based violence measured at T2 was not significantly associated with training discontinuity at T3. These findings suggest that early experiences of sexual orientation-based victimization may disrupt persistence in VET. They underscore the importance of developing inclusive prevention and support strategies aimed at protecting apprentices from sexual orientation-based victimization, with the goal of reducing training discontinuity.

**Supplementary Information:**

The online version contains supplementary material available at 10.1186/s40461-026-00211-0.

## Introduction

Vocational Education and Training (VET) constitutes the most common educational pathway after compulsory schooling in Switzerland. Despite the overall success and strong reputation of the Swiss VET system, not all training trajectories are completed as planned. A considerable proportion of apprentices interrupt their training before completion. National data indicate that approximately one quarter of apprenticeship contracts are terminated prematurely (Bosset et al. 2022).

Previous research on dropout and training discontinuity in VET has primarily focused on structural and individual determinants, such as parental education and self-efficacy (Michaelis and Richter 2022), mismatches between training expectations and workplace realities, insufficient institutional support, or poor working conditions (Böhn and Deutscher 2022). Influential theoretical frameworks, most notably Tinto’s model of academic and social integration (Tinto 1975), emphasize the role of integration processes in shaping educational persistence, yet they have only marginally considered experiences of victimization and discrimination, particularly those related to the gender system^1^. As a result, processes of victimization related to sex, gender identity, and sexual orientation have remained largely peripheral in research on vocational trajectories, despite their potential relevance for feelings of belonging, safety, and continuity.

Drawing on the Minority Stress Model (Meyer 2003), exposure to stigma-related stressors may undermine well-being and persistence by generating chronic stress and perceptions of insecurity (Meyer 2003). Sexual minorities are particularly likely to be exposed to stressful experiences related to discrimination and victimization. In turn, these experiences may contribute to elevated rates of mental health difficulties. Consistent with this perspective, recent empirical evidence suggests that experiences of victimization can also foster intentions to interrupt vocational training (Gianettoni et al. 2025). Building on these considerations, and adopting an exploratory approach, the present study does not aim to test a comprehensive explanatory model of training discontinuity. Rather, it aims to highlight the relevance of considering sex-based and sexual orientation-based victimization when analyzing VET trajectories by examining whether such experiences are longitudinally associated with subsequent training discontinuity among apprentices.

### Sex-based victimization in the VET context

Sex-based victimization refers to experiences of violence directed at an individual on the basis of their administrative sex^2^. This form of victimization remains pervasive within educational settings in Western contexts, including Switzerland. Female students frequently report experiencing various forms of violence based on their assigned sex (Blondé et al. 2025; Chevillard et al. 2016; Leaper and Starr 2019). Women constitute the primary group of victims (WHO, 2021), but, although less common, male students can also be targeted when they transgress traditional masculine norms or fail to conform to dominant models of masculinity (Fleming et al. 2015). Patterns of exposure to sex-based violence vary across educational fields: male-dominated environments tend to present a higher prevalence of harassment and gendered hostility (Dresden et al. 2018; Settles et al. 2006). In these contexts, such as STEM or medical training, women frequently encounter discrimination, stereotyping, and sexual harassment, which contribute to reduced health, well-being and perseverance (Cech and Waidzunas 2011). Also in the VET context, women are exposed to sex-based violence, particularly within male-dominated occupational fields (Blondé et al. 2022; Makarova et al. 2016; Meri et al. 2023).

Research shows that sex-based violence has profound and immediate consequences for victims’ physical safety, psychological well-being, and overall sense of security (Krug et al. 2002), as well as for academic performance (Molstad et al. 2021; Okumu et al. 2020). However, the role of sex-based victimization as a risk factor for training discontinuity or premature training termination in the VET context remains largely unexplored.

### Sexual orientation-based victimization in the VET context

Like sex-based violence, sexual orientation-based victimization remains a reality in educational contexts (Harris et al. 2021; Kosciw et al. 2022; Moyano and Sánchez-Fuentes 2020), including in Switzerland (Blondé et al. 2025; Eisner and Hässler 2021). While victims of sexual orientation-based violence are primarily non-heterosexual students, heterosexual individuals with non-conforming gender expression can also be victims of heterosexist violence, notably because they are perceived as having a non-heterosexual orientation (Déjussel et al. 2024; Lian et al. 2022; Rodríguez-Hidalgo & Hurtado-Mellado 2019).

Unlike other social markers, such as assigned sex, sexual orientation is not outwardly visible. As a result, individuals may choose to conceal their sexual orientation to avoid or minimize exposure to violence (Cohen et al. 2016), particularly those who have previously experienced victimization (Goyer et al. 2015). Concealment has been shown to provide a stronger protective effect in contexts characterized by high levels of homophobia (Pachankis and Bränström 2018). This raises concerns about the potential underestimation of self-reported measures of sexual orientation-based victimization, as some affected individuals may conceal their sexual orientation, particularly in male-dominated fields characterized by strong norms of hegemonic masculinity (Blondé et al. 2022).

Sexual orientation-based victimization has been shown to undermine students’ attention and motivation in school (Chamberland et al. 2013) and is associated with higher rates of truancy and disengagement (Birkett et al. 2014; Burton et al. 2014; Moyano and Sánchez-Fuentes 2020; Poteat et al. 2014). Protective school climates have also been found to reduce the risk of disengagement and dropout (Birkett et al. 2014). Among the few existing longitudinal studies on this topic, Burton et al. (2014) found that, compared to heterosexual youth, non-heterosexual youth reported more excused and unexcused absences, as well as higher levels of depression and anxiety symptoms. Similarly, Sansone (2019), using data from the U.S. High School Longitudinal Study, demonstrated that non-heterosexual youth had lower high-school graduation and college attendance rates. These results were mediated by experiences of sexual orientation-based victimization and by homophobic school climates. Taken together, this body of research suggests that sexual orientation-based victimization, whether directed at openly non-heterosexual individuals or at heterosexual individuals perceived as non-heterosexual, contributes to absenteeism, which in turn lowers graduation rates and may disrupt educational trajectories.

### Our focus

To our knowledge, no longitudinal research has analyzed the impact of both sex-based and sexual orientation-based victimization during the apprenticeship period on the training discontinuity. This leads us to the central question of the present article: to what extent do sex-based or sexual orientation-based forms of victimization during the apprenticeship period influence VET trajectories by facilitating training discontinuity? Addressing this question is crucial given the long-term consequences of training discontinuity for vocational trajectories and the lack of longitudinal evidence regarding the role of sex-based and sexual orientation-based victimization. The findings have implications for theory by extending existing explanations of vocational persistence to account for victimization processes, and for practice by informing policies and support structures aimed at promoting inclusive and stable apprenticeship pathways.

### Current research and hypotheses

The aim of this study, based on a representative sample of apprentices enrolled in vocational training in the canton of Geneva, Switzerland, is to test whether students who experience at least one form of sex-based or sexual orientation-based violence during a given scholar year (Time T) are more likely to experience training discontinuity in the following school year (Time T + 1) compared to their peers who do not experience violence. Analyses controlled for theoretically relevant variables presented above and available in the dataset, namely assigned sex, sex-typing of the field, and concealment of sexual orientation^3^. Although these variables are well-established predictors of exposure to sex- and sexual orientation-based violence, they are also independently associated with vocational training trajectories and risks of training discontinuity. We therefore treated these variables as potential confounders rather than solely as antecedents of violence in order to estimate the association between experiences of violence and subsequent training discontinuity independently of structural and individual characteristics of training pathways.

We hypothesize that:

H1. Sexual orientation-based victimization experienced during the apprenticeship period is positively associated with subsequent training discontinuity.

H1a. Sexual orientation-based victimization reported at Time 1 is positively associated with the probability of training discontinuity between Time 1 and Time 2.

H1b. Sexual orientation-based victimization reported at Time 2 is positively associated with the probability of training discontinuity between Time 2 and Time 3.

H2. Sex-based victimization experienced during the apprenticeship period is positively associated with subsequent training discontinuity.

H2a. Sex-based victimization reported at Time 1 is positively associated with the probability of training discontinuity between Time 1 and Time 2.

H2b. Sex-based victimization reported at Time 2 is positively associated with the probability of training discontinuity between Time 2 and Time 3.

## Materials and methods

### Procedure

This study draws on a longitudinal database. The study population consisted of first-year students enrolled in six vocational training centers (*Centres de formation professionnelle*, CFP) in the canton of Geneva, Switzerland. Within these six institutions, our survey targeted all students enrolled in dual-track programs (as opposed to full-time school-based programs) who began their training in the 2020 academic year. We extended the survey to include all first-year students, regardless of training pathway (dual-track or full-time), primarily to increase the number of female participants in these male-dominated fields^4^. The study includes only vocational learners enrolled in 3‑ or 4‑year VET programs leading to a Federal Certificate of Capacity (CFC). Students enrolled in 2‑year programs were not included.

We conducted the study in the students’ classrooms during the normal school day. Each student was provided with a Qualtrics questionnaire and asked to complete it individually on a computer. The total study time was approximately 30 min and was supervised by the students’ regular teacher, who had been trained to ensure that the instructions were correctly understood and to answer any questions. All responses were fully anonymized. We obtained participants’ consent, as well as parental consent for those under 18, before conducting the study. Ethical approval was obtained from an independent committee of the Geneva Canton Education Research Service and subsequently validated by the Geneva Canton Education Department. Participants did not receive any financial remuneration or incentives for taking part in the study.

### Participants

The questionnaire was administered three times (once per year, between November and February) to all students listed in the first-year class registers in 2020, resulting in a longitudinal sample in which the same individuals were followed across three measurement points. At Wave 1, apprentices had a mean age of 18 years, with 97% being between 14 and 26 years old. Response rates varied across waves: 82% in the first wave, 70% in the second, and 61% in the third, indicating a gradual decline in participation over time.

The analyses focus on the cohort of students who were present in the class registers during the first wave of the study. This cohort was tracked across three time points, from 2020 to 2023. The initial sample consisted of 1,370 students and decreased over time as some students discontinued their training and therefore disappeared from class registers. This study focuses on training discontinuity, measured by absence from the class register.

### Measures

#### Independent variables

*Sexual orientation-based victimization* was measured using a combined indicator assessing whether apprentices had experienced at least one form of violence because of their sexual orientation since the beginning of their apprenticeship (question at Time 1) or since the previous survey (question at Time 2 and Time 3).

To capture the principal forms of gender-based violence described in the literature^5^, four forms of violence were assessed: physical violence (“Have you ever been physically assaulted, e.g., shoved, beaten, or injured, *due to your sexual orientation*?”), verbal violence (“Have you ever been verbally attacked, e.g., insulted, bullied, or mocked, *due to your sexual orientation*?”), psychological violence (“Have you ever been the victim of negative rumors or lies *due to your sexual orientation*?”), and online violence (“Have you ever been the victim of negative rumors or lies via social media or electronic communication *due to your sexual orientation*?”). Response categories included “never,” “at least once,” “at least once every month,” “several times every month,” and “several times every week.” To create a dichotomous indicator distinguishing individuals who had never experienced sexual orientation-based violence from those who had experienced at least one form of such violence, the data were recoded so that all participants were classified as 0 (“never”) if they answered “never” to all four items; otherwise, they were classified as 1 (“at least once”).

This measure was collected at Time 1, Time 2, and Time 3 but was used in the regression models only at Time 1 and Time 2 to predict training discontinuity at Time T + 1.

The indicator of *sex-based victimization* was constructed using the same procedure as that used for sexual orientation-based violence. The questions were identical except that they referred to experiences occurring “*due to your sex*”. This measure was collected at Time 1, Time 2, and Time 3 but was used in the regression models only at Time 1 and Time 2 to predict training discontinuity at Time T + 1.

#### Dependent variable

*Training discontinuity* was the dependent variable and was defined as the *absence of apprentices from class registers in the following academic year (T + 1).*

#### Control variables

##### Assigned sex

Participants were asked to report their sex as indicated on their identification documents (male or female^6^).

S*ex-typing of the field*^7^: Depending on their field of study, respondents were categorized as being enrolled in a male-dominated field (≥ 70% male: CFP Construction and CFP Technical), a female-dominated field (≥ 70% female: CFP Health and CFP Social), or a sex-balanced field (CFP Arts and CFP Commerce).

*Concealment of sexual orientation* was measured using the item: “At school, I try to hide my sexual orientation” (1 = not at all to 6 = absolutely). This measure was collected at Time 1, Time 2, and Time 3 but was used in the regression models only at Time 1 and Time 2 to predict training discontinuity at Time T + 1.

### Analytical strategy

To test our hypotheses, we conducted discrete-time survival analyses using logistic regression models. All analyses were performed in R. Given the longitudinal structure of the data, the dataset was reshaped into a person-period (long) format in which each participant contributed one observation per time point until training discontinuity occurred, or the observation period ended. To account for the substantial amount of missing data, analyses were conducted on 15 multiply imputed datasets, and results were pooled according to Rubin’s rules (see below for details regarding the imputation procedure). It should be noted that, when using imputed datasets, the conventional goodness-of-fit indicators for logistic regression models cannot be computed.

The dependent variable was *training discontinuity*, operationalized as the absence of apprentices from class registers in the following academic year (T + 1). We examined two time points^8^: first, the impact of experiences reported at T1 on presence at T2; second, the impact of experiences reported at T2 on absence at T3. Three regression models were tested. Model 1 included only the independent variables *sexual orientation-based victimization* (during the previous year), *sex-based victimization* (during the previous year), and the effect of time. Model 2 additionally controlled for *assigned sex* and the *sex-typing of the field*. Model 3 further controlled for the *concealment of sexual orientation* during the previous year.

Prior to model estimation, potential multicollinearity among predictors was assessed using the Generalized Variance Inflation Factor (GVIF). The results indicated no evidence of multicollinearity among the predictors (see Supplementary Material, Annex 1).

### Missing data and multiple imputation

The dataset contained a substantial amount of missing data, except for assigned sex and sex-typing of the training field, which were complete because they were supplemented using administrative information from the database of the Department of Education of the Canton of Geneva. All other study variables contained missing values and were therefore included in the imputation procedure.

Analyses of missingness indicated that apprentices who had experienced at least one form of sex-based or sexual orientation-based violence, men, and apprentices enrolled in gender-mixed training fields were significantly overrepresented among cases with missing data^9^ (see Supplementary Material, Annex 2). In addition, missingness was strongly related with apprentices’ presence or absence in the class registers, as data collection could not continue once apprentices had left their class, resulting in structurally missing observations at later waves.

To address missing data, we used multiple imputation, generating and analyzing 15 multiplied imputed datasets (see Supplementary Material, Annex 3 for observed and imputed values). All incomplete variables were imputed using fully conditional specification implemented in the mice package (version 3.16.0) in R (Van Buuren and Groothuis-Oudshoorn 2011). Imputations were performed using predictive mean matching, which preserves the original distribution of the variables and reduces the risk of imputing implausible values (Van Buuren 2018; Heymans and Eekhout 2019).

The imputation model included all variables used in the substantive analyses: assigned sex, sex typing of the field, concealment of sexual orientation, sex-based and sexual orientation-based victimization, and presence/absence in the class registers. Including a broad set of predictors related to both the mechanisms of missingness and the study outcomes increased the plausibility of the Missing at Random (MAR) assumption (Van Buuren 2018).

To assess the quality of the imputations, two key diagnostic checks were conducted. First, because multiple imputation by chained equations relies on an iterative algorithm, trace plots were examined to visually assess the stability of the imputation process across iterations (Van Buuren 2018). Overall, the chains appeared stable, with the different streams intermingling and showing no systematic trends across iterations. Second, the potential scale reduction factor (PSRF) was examined as an additional convergence diagnostic, with values close to 1 indicating satisfactory convergence (Vehtari et al. 2021). However, if missingness depended on unobserved variables, some degree of bias may remain.

Finally, the parameters of interest were estimated separately within each imputed dataset and subsequently combined using Rubin’s pooling rules (Van Buuren 2018).

## Results

### Descriptive statistics

Table 1 presents the descriptive statistics for the study variables across the three measurement waves (T1, T2, and T3). At baseline (T1), the sample included 1,370 apprentices, all of whom were present in the class registers. At T2, 18% of apprentices were absent from the class registers, indicating training discontinuity. This proportion slightly decreased to 15% at T3 among the remaining sample (*N* = 1,122). The distribution of assigned sex remained stable across waves, with approximately two-thirds of the sample identified as men and one-third as women. Similarly, the distribution of sex-typed VET training fields remained consistent over time.

**Table 1:** Descriptive statistics

Variables	T1 (*N* = 1370)	T2 (*N* = 1370)	T3 (*N* = 1122)
Training discontinuity (absence from class register)			
No	1370 (100%)	1122^a^ (82%)	949 (85%)
Yes	0 (0%)	248 (18%)	173 (15%)
Assigned sex			
Men	904 (66%)	904 (66%)	740 (66%)
Women NA’s	465 (34%) 1	465 (34%) 1	382 (34%)
VET sex-typed trainings			
Male-dominated	688 (50%)	688 (50%)	574 (51%)
Sex-balanced	449 (33%)	449 (33%)	361 (32%)
Female-dominated	233 (17%)	233 (17%)	187 (17%)
Sex-based victimization			
Never	1054 (89%)	674 (81%)	495 (80%)
At least 1	132 (11%)	154 (19%)	124 (20%)
NA’s	184	542	503
SO-based victimization			
Never	1125 (94%)	764 (91%)	561 (90%)
At least 1	66 (5.5%)	74 (8.8%)	63 (10%)
NA’s	179	532	498
Concealment of SO			
1 (not at all)	918 (78%)	472 (70%)	350 (70%)
2	147 (12%)	97 (14%)	77 (15%)
3	48 (4.1%)	48 (7.2%)	35 (7.0%)
4	18 (1.5%)	23 (3.4%)	11 (2.2%)
5	20 (1.7%)	10 (1.5%)	7 (1.4%)
6 (completely)	33 (2.8%)	21 (3.1%)	21 (4.2%)
NA’s	186	699	621

^a^ At Time T2, 1122 students were on the class register. Missing data therefore include the 248 students (1370 − 1122) who were no longer eligible to complete the questionnaire. This does not apply to assigned sex and sex-typed field, which are stable across waves and were completed using administrative data

Sex-based victimization (SV) increased over time. While 11% of apprentices reported at least one experience of sex-based violence since the beginning of apprenticeship at T1, this proportion rose to 19% at T2 (experiences occurring since the previous survey at T1) and to 20% at T3 (experiences occurring since the previous survey at T2) among respondents with available data. A comparable pattern was observed for sexual orientation-based victimization, which was reported by 5.5% of apprentices at T1, 8.8% at T2, and 10% at T3^10^. Regarding concealment of sexual orientation, most respondents reported relatively low levels of concealment across all waves.

### Survival analyses

Table 2 presents the results of three logistic regression models, each displayed in a separate column and estimated using 15 multiply imputed datasets. To examine training discontinuity over time, the analyses were restricted to students who remained at risk of discontinuing their training at each time point. Students who had already left the class registers were therefore excluded from subsequent observations. Coefficients are reported as unstandardized log-odds (b) and reflect the association between the predictors and the likelihood of training discontinuity, operationalized as absence from the class registers in the following year (T + 1).

**Table 2:** Logistic regression models predicting training discontinuity at T + 1 from sex-based and sexual orientation-based victimization at time T

Variable	Model 1 b [95% CI]	Model 2 b [95% CI]	Model 3 b [95% CI]
Intercept	-1.52*** [-1.67, -1.37]	-1.50*** [-1.66, -1.35]	-1.58*** [-1.64, -1.52]
Time [Time 1]	-0.18 [-0.43, 0.07]	-0.18 [-0.38, 0.03]	-0.19 [-0.35, -0.01]
Time 1: SO-V [At least 1]	0.82* [0.05, 1.59]	0.81* [0.10, 1.50]	0.75* [0.08, 1.42]
Time 1: Sex-V [At least 1]	-0.32 [-0.69, 0.05]	-0.31 [-0.65, 0.03]	-0.32 [-0.72, 0.09]
Time 2: SO-V [At least 1]	0.24 [-0.18, 0.66]	0.23 [-0.20, 0.65]	0.16 [-0.25, 0.58]
Time 2: Sex-V [At least 1]	-0.09 [-0.41, 0.23]	-0.07 [-0.38, 0.25]	-0.08 [-0.40, 0.23]
Women [Men]		-0.13 [-0.49, 0.23]	-0.13 [-0.49, 0.24]
Sex-balanced [Male-dominated]		0.08 [-0.31, 0.48]	0.08 [-0.31, 0.48]
Female-dominated [Male-dominated]		-0.03 [-0.34, 0.29]	-0.03 [-0.34, 0.29]
Concealment of SO			0.05 [-0.37, 0.46]

Values are unstandardized log-odds coefficients (b). Values in brackets represent 95% confidence intervals. Reference categories are indicated in brackets. *p < .05. **p < .01. ***p < .001

Across all three logistic regression models, sexual orientation-based victimization at Time 1 was positively and significantly associated with training discontinuity at Time 2. Specifically, apprentices who reported at least one experience of sexual orientation-based victimization at Time 1 showed higher odds of being absent from the class registers at the following time point (T + 1; Model 1: *b* = 0.82, 95% CI [0.05, 1.59], *p* = .04). This association remained statistically significant after controlling for administrative sex and sex-typing of the training field (Model 2: *b* = 0.81, 95% CI [0.10, 1.50], *p* = .03), as well as after further adjustment for concealment of sexual orientation (Model 3: *b* = 0.75, 95% CI [0.08, 1.42], *p* = .03). These results provide consistent support for Hypothesis 1a.

In contrast, sexual orientation-based victimization reported at Time 2 was not significantly associated with training discontinuity at Time 3 in any of the models (all *ps* > 0.05), providing no support for Hypothesis 1b.

Similarly, sex-based victimization at both Time 1 and Time 2 was not significantly associated with subsequent training discontinuity at T + 1 across all models (all *ps* > 0.05). Hypothesis 2 was therefore not supported.

Finally, the coefficient for Time, representing the simple effect of time, as well as the coefficients for all control variables—including administrative sex, sex-typing of the training field, and concealment of sexual orientation—were not statistically significant across models (all *ps* > 0.05).

## Discussion

### Sexual orientation-based victimization at the beginning of apprenticeship as a significant predictor of training discontinuity

Our findings indicate that sexual orientation-based victimization at the beginning of the apprenticeship is associated with a significant increase in the log-odds of training discontinuity, operationalized as absence from the class registers in the following year. Consistent with Hypothesis 1a, apprentices who reported at least one form of sexual orientation-based victimization at Time 1 were more likely to be absent from the class register during the following year (T2) compared to their non-victimized peers. The association between sexual orientation-based victimization at Time 1 and training discontinuity at Time 2 remained significant after controlling for assigned sex and sex-typed training fields, indicating that the impact of victimization is not merely a reflection of structural gender segregation within VET. Moreover, even after concealment of sexual orientation was introduced into the model, the association remained significant, underscoring the enduring role of victimization itself as a determinant of training discontinuity. By contrast, sexual orientation-based victimization measured at Time 2 was not significantly associated with training discontinuity at Time 3, providing no support for Hypothesis 1b.

Taken together, this pattern of findings underscores the importance of timing in understanding how victimization shapes vocational trajectories. Drawing on the life course perspective developed by Elder (1998), entry into apprenticeship can be understood as a critical transition period during which trajectories are particularly sensitive to social experiences. At this early stage, roles, expectations, and commitments have not yet stabilized, making apprentices more receptive to contextual signals that shape their assessment of fit and long-term viability within the training environment. In this context, experiences of sexual orientation-based victimization at the beginning of apprenticeship may function as turning points that increase the likelihood of subsequent training discontinuity. Conversely, the absence of a significant effect at later stages suggests that, once trajectories become more consolidated, adverse experiences may have a reduced capacity to redirect established vocational pathways. Moreover, apprentices who remain in training at Time 2 may constitute a selective subgroup of more resilient individuals, either because they have developed effective coping strategies or because those most affected by victimization have already exited the training system.

Our finding that sexual orientation-based victimization at Time 1 predicts training discontinuity extends prior research showing that sexual orientation-based victimization is associated with truancy and school disengagement among secondary school students, including heterosexual individuals perceived as nonconforming to gender norms (Chamberland et al. 2013; Burton et al. 2014; Déjussel et al. 2024; Poteat et al. 2014; Sansone 2019). This finding has several important implications. First, it suggests that efforts aimed at reducing training discontinuity may benefit from greater attention to experiences of sexual orientation-based victimization during apprenticeship, particularly at the beginning of the training pathway. Second, the persistence of the association across different statistical models indicates that the relationship between victimization and training discontinuity is robust even after the inclusion of key controls variables. Consistent with prior research, our findings further suggest that inclusive policies, supportive adults, and explicit anti-discrimination measures may help buffer the negative effects of victimization on student engagement and reduce the risk of disengagement and dropout (Birkett et al. 2014).

### Understanding the non-significant effect of sex-based victimization on training discontinuity

The absence of a significant association between sex-based victimization and training discontinuity contrasts with the consistent and significant effect observed for sexual orientation-based victimization, providing no support for Hypothesis 2. These findings may be tentatively interpreted considering differences in the social and institutional recognition of distinct forms of gender-based violence and discrimination. Sex-based discrimination in education has gained substantial public, institutional, and political visibility over recent decades, particularly through debates surrounding gender equality, women’s access to education, and initiatives aimed at addressing the underrepresentation of women in male-dominated fields such as STEM. This visibility may contribute to the greater social legitimacy of claims related to sex-based victimization, making such experiences more readily acknowledged and addressed.

In contrast, sexual orientation-based victimization remains comparatively less visible and less consistently recognized within VET settings (Guilley et al. 2025). Claims related to sexual orientation may therefore lack comparable legitimacy, potentially limiting access to formal and informal sources of support. As a result, students exposed to sex-based discrimination during apprenticeship may be more likely to mobilize coping resources, seek support, and benefit from existing protective mechanisms within schools than those experiencing sexual orientation-based victimization during apprenticeship. In this context, the absence of a significant association between sex-based victimization and training discontinuity may reflect the presence of buffering factors rather than the absence of discriminatory experiences. Future research is needed to empirically examine this interpretation.

### Limitations and future research

Although the present study offers novel insights into the relationship between group-based victimization and training discontinuity, several limitations should be acknowledged.

First, the set of control variables included in the analyses was limited by data availability. While we adjusted for key structural characteristics theoretically relevant to both victimization and training trajectories (administrative sex, sex-typing of the training field, and concealment of sexual orientation), other factors commonly included in models of educational persistence (e.g., prior academic performance, socioeconomic background, mental health, and family resources) were not available. The absence of these variables limits both causal interpretation and internal validity. Future research should build on these findings using richer datasets and more explicit theoretical models to strengthen both internal and external validity.

Second, the outcome measure captures *training discontinuity* operationalized as absence from class registers in the subsequent year rather than formally verified premature training termination. Although absence from the registers constitutes a meaningful indicator of instability in training trajectories, it does not allow us to distinguish among different situations, such as contract termination, grade repetition, delayed progression, or continuation of training in another canton or institution. Distinguishing among these pathways would contribute to a more precise understanding of the educational consequences of victimization. Future studies linking administrative and contractual data would be particularly valuable.

Third, concealment of sexual orientation was measured only in relation to the school context. Because apprentices alternate between school-based and workplace settings, concealment strategies may vary substantially across contexts. Our measure therefore provides only a partial representation of concealment processes during apprenticeship and may underestimating both their complexity and their role in shaping exposure to victimization and training outcomes. Future research should assess concealment across multiple contexts.

Fourth, the analyses relied on self-reported measures of victimization and concealment. Prior research indicates that disclosure decisions can influence both the likelihood of reporting victimization and the perceived severity of these experiences (Pachankis and Bränström 2018). Consequently, the prevalence of sexual orientation–based victimization may be underestimated in this study, particularly in strongly male-dominated training fields, where heterosexist norms may discourage disclosure and reporting. This limitation should be considered when interpreting prevalence estimates and group differences.

Fifth, the study does not include measures of victimization based on gender identity or gender expression. This omission is important because existing research demonstrates that non-cisgender individuals and those who do not conform to traditional gender expressions are at heightened risk of heterosexist victimization (Lian et al. 2022). Future research should explicitly incorporate these dimensions, in addition to sex-based victimization, to more comprehensively capture the impact of victimization related to the gender system on vocational training trajectories.

To better understand the differential impacts of sex-based and sexual orientation-based victimization, as well as the ways in which these impacts evolve over time, future research should integrate victimization into more comprehensive longitudinal models that include the individual, institutional, and contextual determinants theorized in the literature on dropout from initial vocational training (Böhn and Deutscher 2022). In addition, greater attention should be paid to protective and buffering mechanisms, such as supportive peers, teachers, parents, and inclusive institutional policies. Examining how these resources contribute to resilience may help clarify why some forms of victimization are more strongly associated with training discontinuity than others.

## Conclusion

This longitudinal study demonstrates that experiences of sexual orientation-based victimization at the beginning of an apprenticeship significantly predict training discontinuity. The effect remained significant across multiple models, even after controlling for assigned sex, sex-typing of training fields, and concealment of sexual orientation. These findings highlight the persistent and detrimental role of sexual orientation-based victimization during the apprenticeship period in shaping students’ educational trajectories. They also extend previous research on dropout and training discontinuity by incorporating theses underexplored predictors.

## Supplementary Information

Below is the link to the electronic supplementary material.

Supplementary Material 1.

Supplementary Material 2.

## Data Availability

The datasets used and analyzed during the current study are available from the corresponding author on reasonable request.

## Abbreviation

VET: Vocational Education and Training

## Footnotes

1 Following Connell (1987), the gender system refers to a hierarchical social structure in which relations of sex, gender, and sexuality are jointly organized through power relations, with heterosexuality embedded as a normative and regulatory principle.

2 In this study, the term *sex* refers to administrative sex, as recorded in official identity documents. This concept is analytically distinct from gender identity (e.g., cisgender or transgender women and men). As the present data do not include specific indicators of gender identity, our analyses are limited to victimization based on administrative sex (in Switzerland, women vs. men).

3 The dataset did not include additional variables (like performance during training or migration background, see Böhn and Deutscher 2022) that would have allowed further adjustment in the models.

4 The study was initially designed to focus exclusively on dual vocational training. However, to increase the representation of girls in male-dominated fields, we also included full-time training programs. In these fields, girls are more frequently enrolled in full-time vocational education than in dual training.

5 Following Krug et al. (2002), violence is traditionally categorized into physical, verbal, and psychological. In our work, we also include online violence, such as harassment and abuse on social media, which has become increasingly prevalent in contemporary society (Donato et al. 2022).

6 Switzerland recognizes only 2 sexes, male and female. Missing data were completed using the database of the Educational Research Service (SRED) of the Canton of Geneva.

7 In line with previous research, we consider training programs to be sex-typed if the sex-ratio exceeds 70%, and sex-balanced otherwise (Imdorf and Hupka-Brunner 2015). This categorization reflects the sex segregation that persists in Switzerland’s VET system. Missing data were completed using the database of the Department of Education of the Canton of Geneva.

8 No data were available for T4.

9 We conducted Pearson’s chi-squared tests for each categorical variables and examined the associated standardized residuals to assess whether missingness was systematically related to observed characteristics. Regarding the concealment of sexual orientation, we conducted a Wilcoxon test, showing no significant difference between the complete and incomplete groups.

10 Detailed descriptive statistics by specific type of victimization are available in the Supplementary Material (Annex 4).
