# Supplementary material for: Impact of sex-based and sexual orientation-based victimization on training discontinuity among swiss apprentices: a longitudinal study
Source: Empir Res Vocat Educ Train. 2026 Jun 20;18(1):7. doi: 10.1186/s40461-026-00211-0 (PMC13283172; doi:10.1186/s40461-026-00211-0)

Supplementary material 1: Standardized residuals from chi-squared tests

|  | Complete case | |
| --- | --- | --- |
|  | FALSE | TRUE |
| **VET sex-typed trainings** |  |  |
| Male-dominated | -1.756499 | 1.756499 |
| Gender balanced | 3.216869 | -3.216869 |
| Female-dominated | -1.681461 | 1.681461 |
| **Assigned Sex** |  |  |
| Men | 2.012225 | -2.012225 |
| Women | -2.012225 | 2.012225 |
| **Time** |  |  |
| T1 | -25.63970 | 25.63970 |
| T2 | 7.411478 | -7.411478 |
| T3 | 18.228229 | -18.228229 |
| **Presence in class registers** |  |  |
| Yes | -31.84898 | 31.84898 |
| No | 31.84898 | -31.84898 |
| **SO based Violence** |  |  |
| Never | -3.90283 | 3.90283 |
| At least 1 | 3.90283 | -3.90283 |
| **Sex-based violence** |  |  |
| Never | -2.565372 | 2.565372 |
| At least 1 | 2.565372 | -2.565372 |

To assess the distribution of missing data across the dataset, we examined the association between each variable and a missingness indicator, which takes the value *TRUE* when a record (row) is fully observed and *FALSE* otherwise. We conducted Pearson's chi-squared tests for each variable and interpreted the standardised residuals to evaluate whether the presence of missing data was systematically related to some observed categories. Values exceeding ±1.96 at the 0.05 significance level mean that a specific category is significantly over- or underrepresented among missing data. Regarding the concealment of sexual orientation, we conducted a Wilcoxon test, showing no significant difference between the complete and incomplete groups (p-value = 0.29).

Supplementary material 2: Comparing observed (black) and imputed (blue bars) values for **sex-based violence** (top) and **SO-based violence** (bottom) – T1, T2, T3


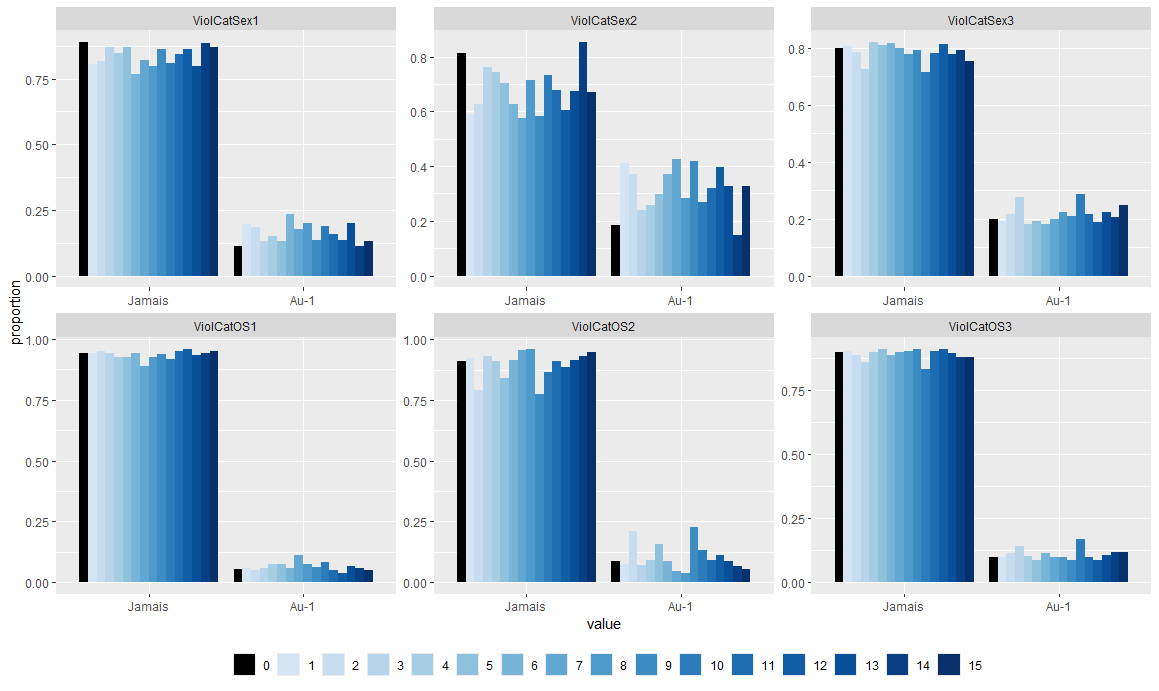


2.1. Densityplot of observed (blue) and imputed (red) values for the **concealment of sexual orientation** – T1, T2, T3


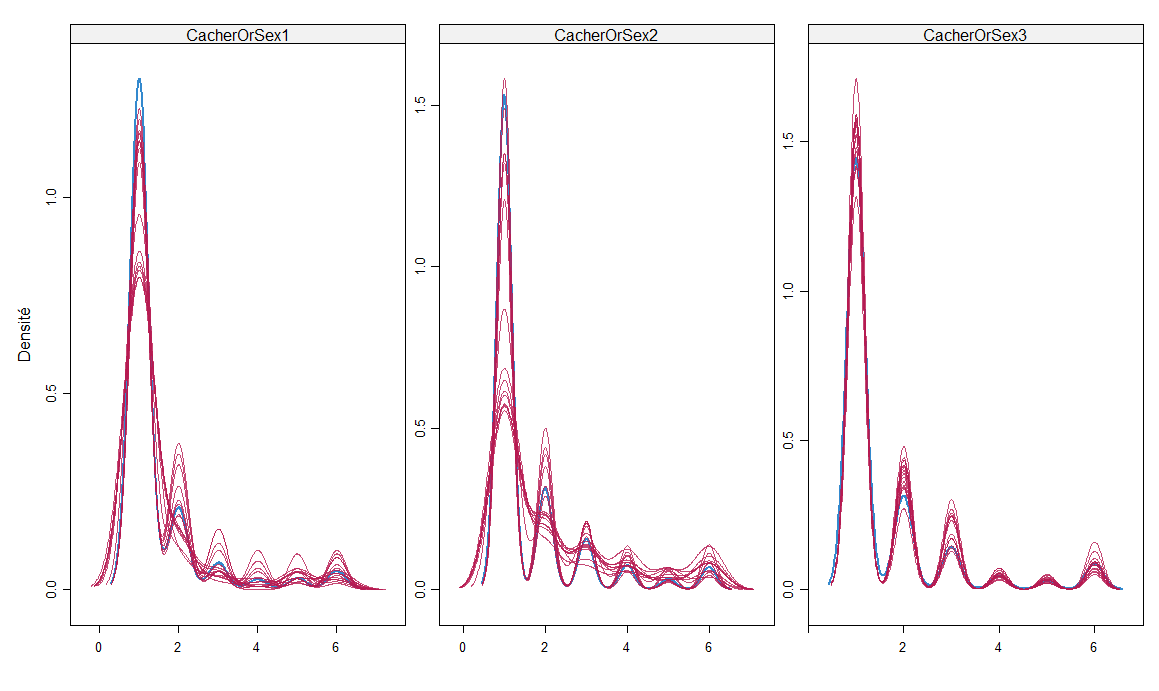


Supplementary material 3 : Influx and outflux measures
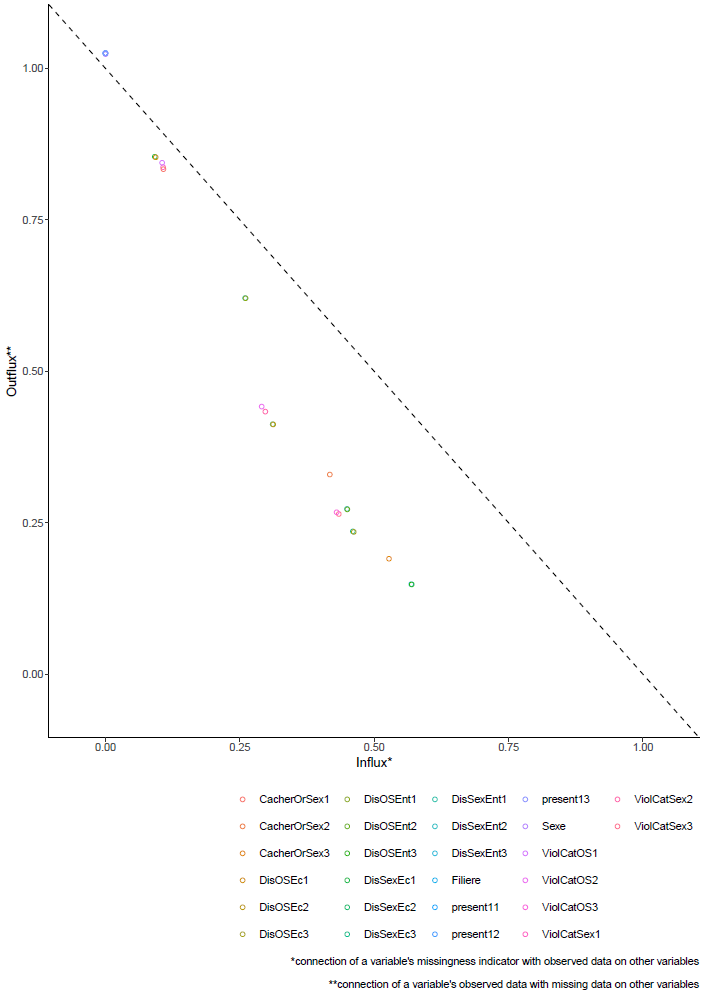


Supplementary material 4 : Diagnostics of the imputations

4.1. Convergence

Mice imputations rely on an iterative Markov Chain Monte Carlo (MCMC) algorithm. The following traceplots can be used to visually assess the convergence of MCMC chains through iterations (Van Buuren, 2018). Overall, the convergence of our chains is good, with the different streams intermingling freely and showing no definite trends through iterations.


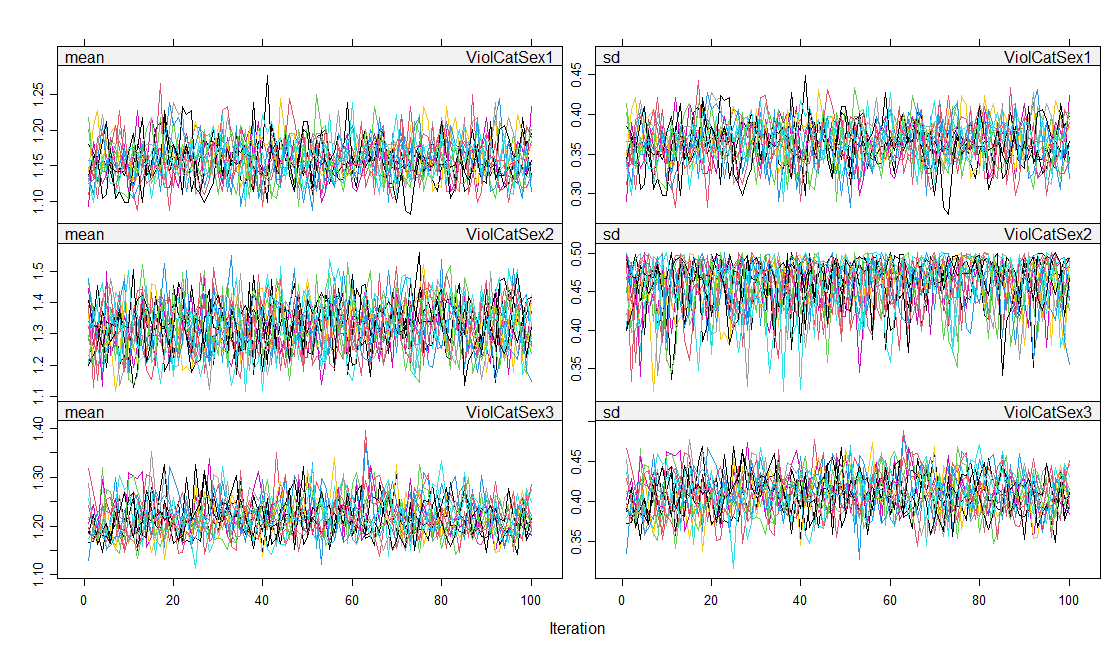


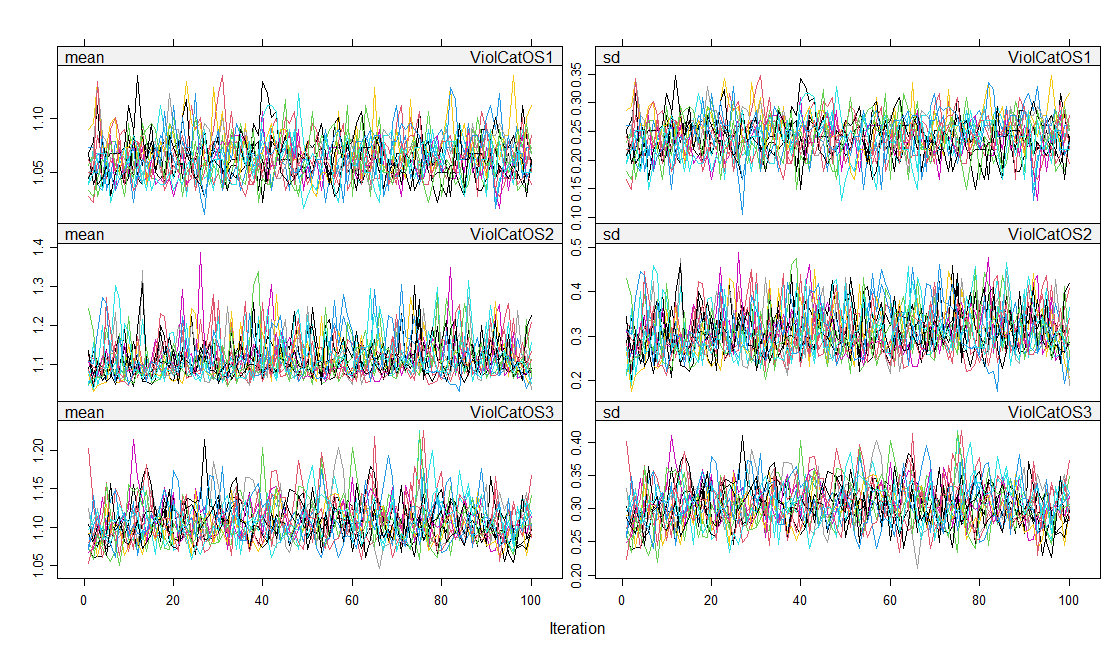


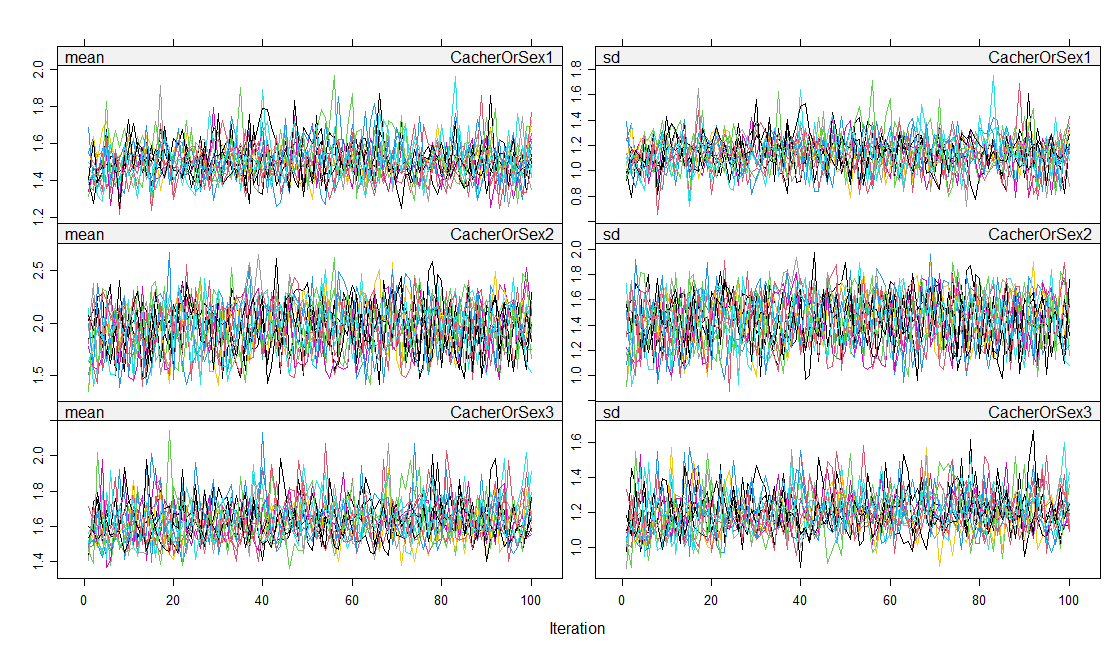


4.2. Potential scale reduction factor through iterations

The second key diagnostic for the MCMC convergence is the potential scale reduction factor, which is one of the most widely used convergence diagnostics (Gelman and Rubin, 1992; Vehtari *et.al.*, 2021). As can be seen in the plot below, our chains have reached good convergence, with PSRF values close to 1.


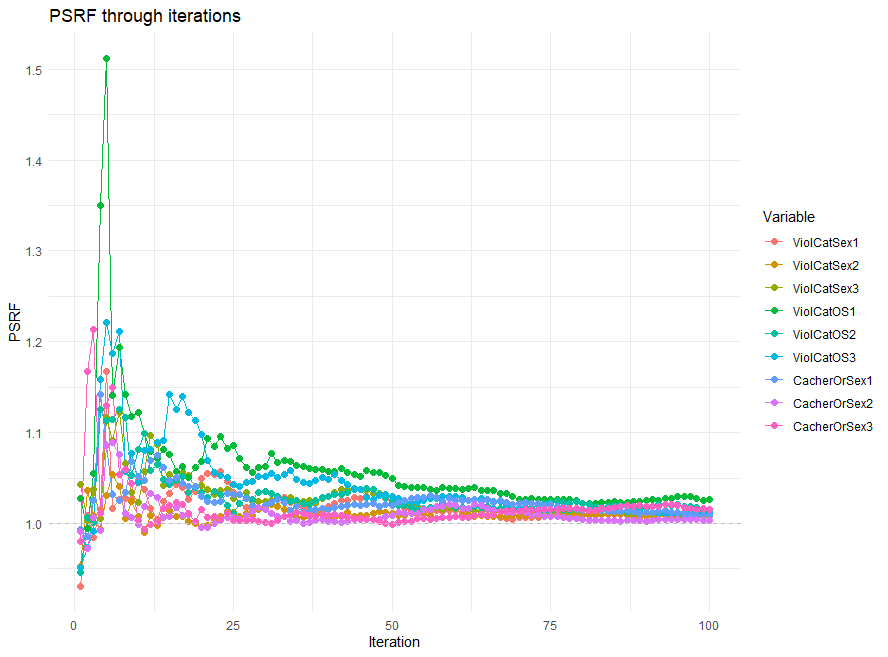

Supplement: Supplementary file 1 — Supplementary Material 1. [file 40461_2026_211_MOESM1_ESM.docx]
