# Supplementary material for: Impact of sex-based and sexual orientation-based victimization on training discontinuity among swiss apprentices: a longitudinal study
Source: Empir Res Vocat Educ Train. 2026 Jun 20;18(1):7. doi: 10.1186/s40461-026-00211-0 (PMC13283172; doi:10.1186/s40461-026-00211-0)
Supplement: Supplementary file 2 — Supplementary Material 2. [file 40461_2026_211_MOESM2_ESM.docx]

**Supplementary material 1: Detect potential multicolinearity problems**

|  | GVIF | Df | GVIF^(1/(2*Df)) |
| --- | --- | --- | --- |
| Time | 1.015697 | 1 | 1.007817 |
| SO-based victimization (SO-V) | 1.411072 | 1 | 1.187846 |
| Sex-based victimization (Sex-V) | 1.366965 | 1 | 1.169099 |
| Assigned sex | 1.490852 | 1 | 1.221000 |
| VET sex-typed trainings | 1.463881 | 2 | 1.099958 |
| Concealment of SO | 1.118364 | 1 | 1.057504 |

To detect potential multicollinearity problems, we estimated the Generalized Variance Inflation Factor (GVIF) for each predictor included in the logistic regression model, pooled across the 15 imputed datasets. We report GVIF^(1/(2·Df)) to allow comparison across predictors with different degrees of freedom. As shown in Table X, all values are close to 1 and well below the conventional threshold of 2, indicating no evidence of multicollinearity among predictors.

**Supplementary material 2: Standardized residuals from chi-squared tests**

|  | Complete case | |
| --- | --- | --- |
|  | FALSE | TRUE |
| **VET sex-typed trainings** |  |  |
| Male-dominated | -1.756499 | 1.756499 |
| Gender balanced | 3.216869 | -3.216869 |
| Female-dominated | -1.681461 | 1.681461 |
| **Assigned Sex** |  |  |
| Men | 2.012225 | -2.012225 |
| Women | -2.012225 | 2.012225 |
| **Time** |  |  |
| T1 | -25.63970 | 25.63970 |
| T2 | 7.411478 | -7.411478 |
| T3 | 18.228229 | -18.228229 |
| **Presence in class registers** |  |  |
| Yes | -31.84898 | 31.84898 |
| No | 31.84898 | -31.84898 |
| **SO based Victimization** |  |  |
| Never | -3.90283 | 3.90283 |
| At least 1 | 3.90283 | -3.90283 |
| **Sex-based Vicitmization** |  |  |
| Never | -2.565372 | 2.565372 |
| At least 1 | 2.565372 | -2.565372 |

To assess the distribution of missing data across the dataset, we examined the association between each variable and a missingness indicator, which takes the value *TRUE* when a record (row) is fully observed and *FALSE* otherwise. We conducted Pearson's chi-squared tests for each variable and interpreted the standardised residuals to evaluate whether the presence of missing data was systematically related to some observed categories. Values exceeding ±1.96 at the 0.05 significance level mean that a specific category is significantly over- or underrepresented among missing data. Regarding the concealment of sexual orientation, we conducted a Wilcoxon test, showing no significant difference between the complete and incomplete groups (p-value = 0.29).

**Supplementary material 3: Comparing observed (black) and imputed (blue bars) values for sex-based victimization (top) and SO-based victimization (bottom) – T1, T2, T3**


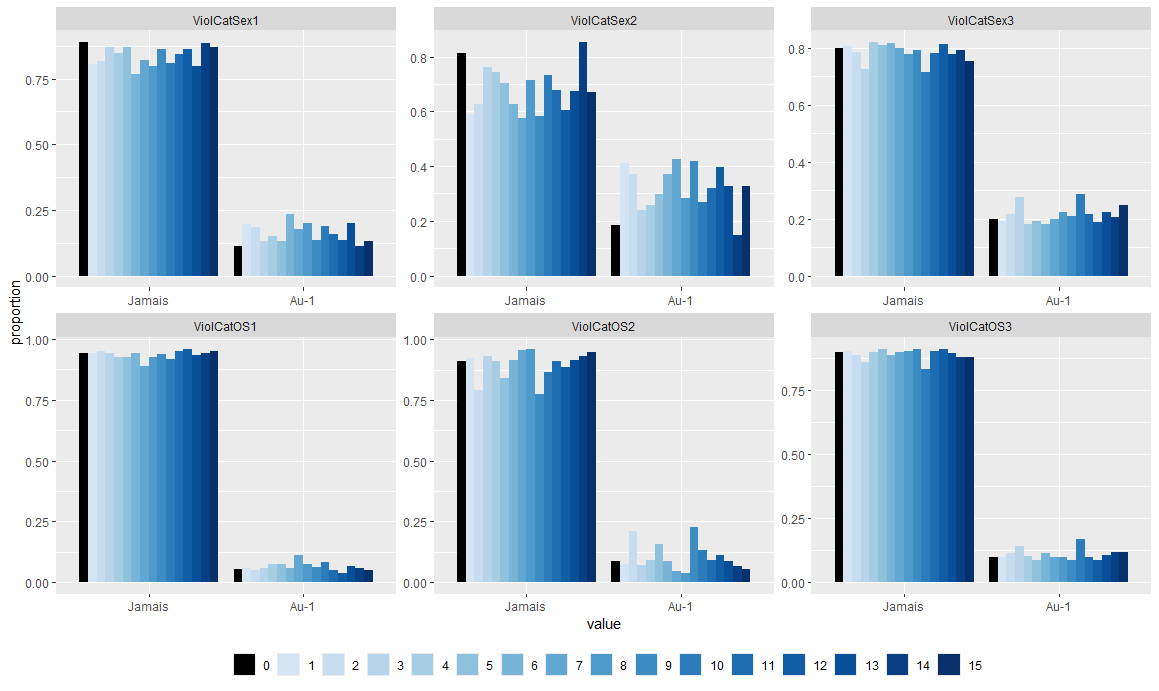


3.1. Densityplot of observed (blue) and imputed (red) values for the **concealment of sexual orientation** – T1, T2, T3


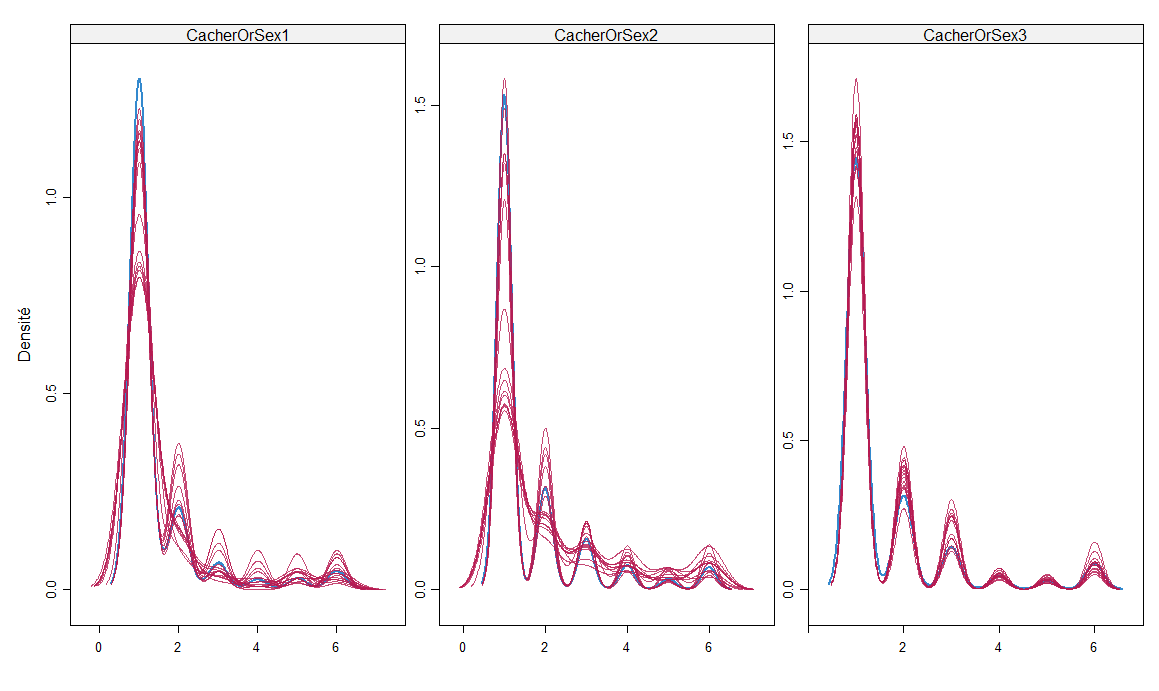


**Annex 4 :** **Frequency results for the four forms of violence for sex-based and so-based victimization**

ViolCat1(physical violence due to sex)

|  | Time 1 | Time 2 | Time 3 |
| --- | --- | --- | --- |
| Non (0) | 1167 | 965 | 784 |
| Oui (1) | 37 | 93 | 80 |
| NA | 533 | 679 | 873 |

ViolCat2 (physical violence due to sexual orientation)

|  | Time 1 | Time 2 | Time 3 |
| --- | --- | --- | --- |
| Non (0) | 1177 | 1009 | 813 |
| Oui (1) | 21 | 48 | 49 |
| NA | 539 | 680 | 875 |

ViolCat3 (verbal violence due to sex)

|  | Time 1 | Time 2 | Time 3 |
| --- | --- | --- | --- |
| Non (0) | 1117 | 936 | 738 |
| Oui (1) | 82 | 119 | 124 |
| NA | 538 | 682 | 875 |

ViolCat4 (verbal violence due to sexual orientation)

|  | Time 1 | Time 2 | Time 3 |
| --- | --- | --- | --- |
| Non (0) | 1155 | 1002 | 804 |
| Oui (1) | 43 | 53 | 58 |
| NA | 539 | 682 | 875 |

ViolCat5 (psychological violence due to sex)

|  | Time 1 | Time 2 | Time 3 |
| --- | --- | --- | --- |
| Non (0) | 1143 | 963 | 789 |
| Oui (1) | 54 | 92 | 72 |
| NA | 540 | 682 | 876 |

ViolCat6 (psychological violence due to sexual orientation)

|  | Time 1 | Time 2 | Time 3 |
| --- | --- | --- | --- |
| Non (0) | 1164 | 996 | 801 |
| Oui (1) | 29 | 59 | 59 |
| NA | 544 | 682 | 877 |

ViolCat7 (online violence due to sex)

|  | Time 1 | Time 2 | Time 3 |
| --- | --- | --- | --- |
| Non (0) | 1163 | 991 | 799 |
| Oui (1) | 19 | 26 | 27 |
| NA | 555 | 720 | 911 |

ViolCat8 (online violence due to sexual orientation)

|  | Time 1 | Time 2 | Time 3 |
| --- | --- | --- | --- |
| Non (0) | 1179 | 1010 | 805 |
| Oui (1) | 20 | 43 | 52 |
| NA | 538 | 684 | 880 |
